# Vedolizumab plus basiliximab as second-line therapy for steroid-refractory lower gastrointestinal acute graft-versus-host disease

**DOI:** 10.3389/fimmu.2024.1408211

**Published:** 2024-07-03

**Authors:** Zicheng Gao, Zhiping Fan, Zhi Liu, Xu Ye, Yunxin Zeng, Li Xuan, Fen Huang, Ren Lin, Jing Sun, Qifa Liu, Na Xu

**Affiliations:** ^1^ Department of Hematology, Nanfang Hospital, Southern Medical University, Guangzhou, China; ^2^ Department of Hematology, Guangdong Second Provincial General Hospital, Guangzhou, China; ^3^ Department of Hematology, Second Affiliated Hospital of Guangzhou Medical University, Guangzhou, China; ^4^ Department of Hematology, The Seventh Affiliated Hospital of Sun Yat-Sen University, Shenzhen, China

**Keywords:** HSCT, vedolizumab, basiliximab, steroid-refractory GVHD, lower intestinal GVHD, α4β7

## Abstract

**Background:**

Steroid-resistant (SR) lower gastrointestinal (LGI) tract graft-versus-host disease (GVHD) is the predominant cause of morbidity and mortality from GVHD after allogeneic hematopoietic stem cell transplantation (allo-HSCT). The role of vedolizumab in the treatment of SR-LGI acute GVHD (aGVHD) remains uncertain. We aimed to assess the efficacy and safety of vedolizumab combined with basiliximab as second-line therapy for SR-LGI-aGVHD.

**Methods:**

This study aimed to explore the efficacy of vedolizumab combined with basiliximab for SR-LGI-aGVHD. The primary endpoint was the overall response (OR) on day 28. Secondary and safety endpoints included durable OR at day 56, overall survival (OS), chronic GVHD (cGVHD), non-relapse mortality (NRM), failure-free survival (FFS), and adverse events.

**Results:**

Twenty-eight patients with SR-LGI-aGVHD were included. The median time to start of combination therapy after SR-LGI-aGVHD diagnosis was 7 (range, 4–16) days. The overall response rate (ORR) at 28 days was 75.0% (95% CI: 54.8%–88.6%), and 18 achieved a complete response (CR) (64.3%, 95% CI: 44.1%–80.7%). The durable OR at day 56 was 64.3% (95% CI: 44.1%–80.7%). The 100-day, 6-month, and 12-month OS rates for the entire cohort of patients were 60.7% (95% CI: 45.1%–81.8%), 60.7% (95% CI: 45.1%–81.8%), and 47.6% (95% CI: 31.4%–72.1%), respectively. The median failure-free survival was 276 days; (95% CI: 50–not evaluable) 12-month NRM was 42.9% (95% CI: 24.1%–60.3%). The 1-year cumulative incidence of cGVHD was 35.7%. Within 180 days after study treatments, the most common grade 3 and 4 adverse events were infections. Nine (32.1%) patients developed cytomegalovirus (CMV) reactivation complicated with bacterial infections (25.0%, CMV infection; 7.1%, CMV viremia). Epstein–Barr virus (EBV) reactivation occurred in five patients (17.9%, 95% CI: 6.8%–37.6%). Only three patients (10.7%, 95% CI: 2.8%–29.4%) in our study developed pseudomembranous colitis.

**Conclusions:**

Vedolizumab plus basiliximab demonstrated efficacy in severe SR-LGI-aGVHD and was well-tolerated. Vedolizumab plus basiliximab may be considered a potential treatment option for patients with LGI-aGVHD.

## 1 Introduction

Lower gastrointestinal (LGI) graft-versus-host disease (GVHD) remains one of the major obstacles to the success of allogeneic hematopoietic stem cell transplantation (allo-HSCT) (1). Currently, corticosteroids remain the standard first-line treatment for acute GVHD (aGVHD); however, the response rate is only in approximately 50% of patients, and long-term prognosis is extremely poor for those with steroid-resistant (SR) aGVHD (2–4). Moreover, the involvement of the lower GI has been reported to be one of the significant independent risk factors for glucocorticoid resistance, and the long-term survival rate for patients with severe aGVHD (grades III–IV) of the lower GI tract is only 25% (5). Second- and third-line treatments are less than optimally documented because of the imbalances of fluid caused by secretory diarrhea, the high risk of severe bleeding, especially if concomitant thrombocytopenia is present, and the frequent overlap of multiple infections in patients with immunocompromised conditions. The treatment of SR-LGI-GVHD is particularly challenging.

Vedolizumab is a monoclonal antibody that specifically targets α4β7 integrin, inhibits its adhesion to mucosal addressing cellular adhesion molecule1 (MAdCAM-1), and selectively blocks gut lymphocyte trafficking without interfering with trafficking to the central nervous system (6). It was approved by the Food and Drug Administration for the treatment of moderate-to-severe inflammatory bowel diseases (IBDs) (7, 8). It has been well established that expression of α4β7 on donor T cells has been shown to be important in the development of intestinal GVHD compared with patients with skin GVHD and those without GVHD (9–13). Recently, phase 1b data (14) suggest that administered vedolizumab for GVHD prevention exhibited a lower incidence of intestinal aGVHD and was well-tolerated. However, conflicting data (15–20) have emerged in the last few years on the efficacy of vedolizumab for the treatment of GI-SR-GVHD. Series studies showed that clinical response varied from 35% to 79% (15, 17, 19) in GI-SR-GVHD treatment. Through a variety of inflammatory and signaling pathways involved in the development of GVHD, topically blocking α4β7/MAdCAM-1 axis may be insufficient. Basiliximab is a monoclonal antibody that inhibits the interleukin-2 receptor (IL-2R) on activated lymphocytes, thereby preventing aGVHD by blocking the effect of IL-2 (21). At present, basiliximab is a common second-line therapy for aGVHD that has shown encouraging results in previous studies (22, 23).

We hypothesized that dual IL-2R and α4β7/MAdCAM-1 axis blockade may further improve the clinical outcomes of patients with SR-LGI-GVHD and have an acceptable level of infection. Thus, we retrospectively analyzed the efficacy and safety of vedolizumab combined with basiliximab as a specified second-line therapy for SR-LGI-aGVHD.

## 2 Methods

### 2.1 Study design and patients

We retrospectively analyzed the efficacy and safety of vedolizumab combined with basiliximab for SR-GI-aGVHD at four hospitals in China (Nanfang Hospital, Guangdong Second Provincial General Hospital, the Second Affiliated Hospital of Guangzhou Medical University, and the Seventh Affiliated Hospital of Sun Yat-Sen University). Inclusion criteria were any patient 18 years old who had undergone allo-HSCT using any donor or graft source, with any conditioning regimen between March 2019 and March 2022.

The diagnostic criteria for aGVHD are based on the literature criteria established by the Mount Sinai Acute GVHD International consortium group. SR aGVHD was defined as disease progression after 3 days of therapy onset with ≥2 mg kg^−1^ day^−1^ of systemic glucocorticoid or equivalent, or lack of improvement after 7 days of treatment initiation, or failure during methylprednisolone taper. This study was approved by the institutional review board of each participating hospital, and all patients (or their guardians) provided signed written informed consent before inclusion. The study was conducted in accordance with the Declaration of Helsinki.

### 2.2 Treatment

Patients received methylprednisolone as the first-line treatment at a dose of 1 mg/kg. If the patients were diagnosed with SR aGVHD, combination therapy was started with basiliximab (20 mg per dose on days 1, 3, and 8 and repeated weekly until aGVHD was improved to grade < II) and vedolizumab. Patients were administered vedolizumab at the same dosage and schedule and approved for the therapy of IBD: an initial dose of 300 mg IV on days 1, 15, 43, and 71. Whether to continue vedolizumab was based on the response evaluated on day 28. In addition to cyclosporine A (CSA) and mycophenolate mofetil (MMF), no other immunosuppressants were administered during the vedolizumab–basiliximab treatment. CSA and MMF were initiated on day −9.

Patients with complete response (CR) and no response (NR) discontinued vedolizumab and basiliximab, but the CSA and MMF were continued, while patients with partial response (PR) continued to receive vedolizumab until aGVHD showed CR or vedolizumab was received for four courses. Supportive care followed department guidelines for each patient. Patients without prior fungal infection were administered fluconazole as prophylaxis against fungal infection, while patients with previous fungal infections received treatment with previously effective antifungal drugs. Steroids were gradually tapered by 30% every 5 days and stopped within 4 weeks after the second dose of basiliximab. Other immunosuppressants, such as MMF, methotrexate (MTX), ruxolitinib and mammalian target of rapamycin (mTOR) inhibitor, mesenchymal stromal cells (MSCs), and fecal microbiota transplantation (FMT), were allowed in NR patients.

### 2.3 Assessments

All patients were diagnosed as LGI-aGVHD by pathological biopsy. aGVHD was staged and graded according to the consensus criteria, and the assessment of response was evaluated by standard definitions. CR was defined as complete remission and improvement of aGVHD signs and symptoms. PR was defined as an improvement of one stage in lower GI aGVHD without any additional systemic treatment, while other organs showed no progression. NR was defined as the absence of remission in lower GI aGVHD without progression in any other organ. Progression was defined as the deterioration in one or more organs by one or more stages and without improvement in any involved GVHD organ. Patients who died before day 28 were considered non-responders, and those who had worsened underlying disease before day 28 were considered non-evaluable.

Safety analysis was conducted by reviewing adverse events (AEs) and malignancy recurrence in all patients through medical records. AEs were recorded between the first dose of the combination and the last follow-up, including infusion reactions, infection, hepatotoxicity, or progressive multifocal leukoencephalopathy (PML). Follow-up care was conducted through physical examination and laboratory assessments, such as routine blood testing, biochemical tests, bone marrow assessment, and pathogen detection [cytomegalovirus (CMV) DNA, Epstein–Barr virus (EBV) DNA, etc.].

Assessments of AEs were performed daily for the first week, weekly from week 1 to week 8, monthly from the second month to the third month, and every 3 months thereafter to collect data on progression, survival, chronic GVHD (cGVHD), and safety outcomes including malignancy relapse and infections.

### 2.4 Endpoints

The primary endpoint was the overall response at day 28 (time from combination treatment to day 28), which was defined as the proportion of patients who had a CR or PR as compared with baseline organ staging. The key secondary endpoint was the duration of overall response at day 56, which was defined as the proportion of patients who had a CR or PR at day 28 and maintained response until day 56. Other secondary endpoints included failure-free survival (FFS) (time from combination to GVHD relapse or malignancy recurrence, non-relapse related death, or the addition of new systemic therapy for aGVHD), overall survival (OS), the onset of cGVHD [according to the National Institutes of Health (NIH) criteria], and non-relapse mortality (NRM).

### 2.5 Statistical analysis

The data cutoff was October 30, 2022. Complete response rate (CRR), overall response rate (ORR), and two-sided 95% confidence intervals (CIs) were calculated using the Wilson method. Fisher’s exact test was used for categorical variables and the Mann–Whitney U tests for continuous variables. Kaplan–Meier (KM) methodology was used to estimate FFS and OS, R was used for plotting the KM curve, and the hazard ratios (HRs) were also calculated, together with its 95% CI, using a stratified Cox model. All statistical tests were two-tailed with a significance level of 0.05. SPSS version 20.0 and R version 3.3.0 were used for data analysis.

## 3 Result

### 3.1 Patient characteristics

Between March 2019 and March 2022, a total of 28 patients from four hospitals were included in this study. There were nine (32.1%) women and 19 (67.9%) men, with a median age of 36 years (range, 18 to 58 years). Acute leukemia was the most common indication for allo-HSCT. Sixteen (57.1%) patients received transplants from an HLA-haploidentical donor (HID) and 12 (42.8%) patients from an HLA-matched sibling donor (MSD). CsA was used in combination with both MTX and MMF as aGVHD prophylaxis for all patients; patients with HID were administered antithymocyte globulin (ATG). Prior to starting basiliximab and vedolizumab, all patients had grade III (n = 13, 46.4%) to IV (n = 15, 53.6%) LGI-aGVHD, only one patient had aGVHD of the LGI tract alone, 24 (85.7%) involved liver aGVHD, and 14 (50.0%) involved skin aGVHD. Diagnosis of all patients was confirmed by colonoscopy biopsy. The median exposure to glucocorticoid for all patients before combination treatment was 6 days (range, 3–15). All patients received the combination of basiliximab and vedolizumab as second-line therapy after failing to respond to glucocorticoid. The median days to start of combination treatment after GI aGVHD diagnosis was 7 days (range, 4–16). The median dose of basiliximab given was 3 (range, 1–5). The median dose of vedolizumab given was 2 (range, 1–3). The median follow-up was 13 months for all patients. The full details of the baseline are given in **Table 1**.

**Table 1:** Characteristics of the patients at baseline.

Characteristics		Number of patients (n = 28)
Gender		
Male		19 (67.9%)
Female		9 (32.1%)
Age		
Median		38 (range, 18–58)
Diagnosis		
AML		9 (32.1%)
ALL		10 (35.7%)
MDS		7 (25.0%)
CMML		1 (3.6%)
PMF		1 (3.6%)
Donor source		
Peripheral blood		28 (100%)
Bone marrow		0
Type of donor		
MSD		12 (42.8%)
HID		16 (57.1%)
GVHD prophylaxis		
MTX + CsA + MMF		28 (100%)
ATG		16 (57.1%)
LGI GVHD stage		
III		13 (46.4%)
IV		15 (53.6%)
GVHD site(s)		
Skin		14 (50.0%)
Liver		24 (85.7%)
Time to aGVHD, days		
Median		35 (range, 15–158)
Time to vedolizumab after aGVHD, median days (range)		7 days (range, 4–16)
Number of doses of vedolizumab, median, (range)		2 (range, 1–3)
Number of doses of basiliximab, median, (range)		3 (range, 1–5)

AML, acute myelogenous leukemia; ALL, acute lymphoid leukemia; MDS, myelodysplastic syndromes; CMML, chronic myelomonocytic leukemia; PMF, primary myelofibrosis; GVHD; graft-versus-host disease; MSD, HLA-matched sibling donor; HID, HLA-haploidentical donor; MTX, methotrexate; CsA, cyclosporine A; MMF, mycophenolate mofetil; ATG, antithymocyte globulin; LGI, lower gastrointestinal.

### 3.2 Treatment response and survival

**Figure 1:**
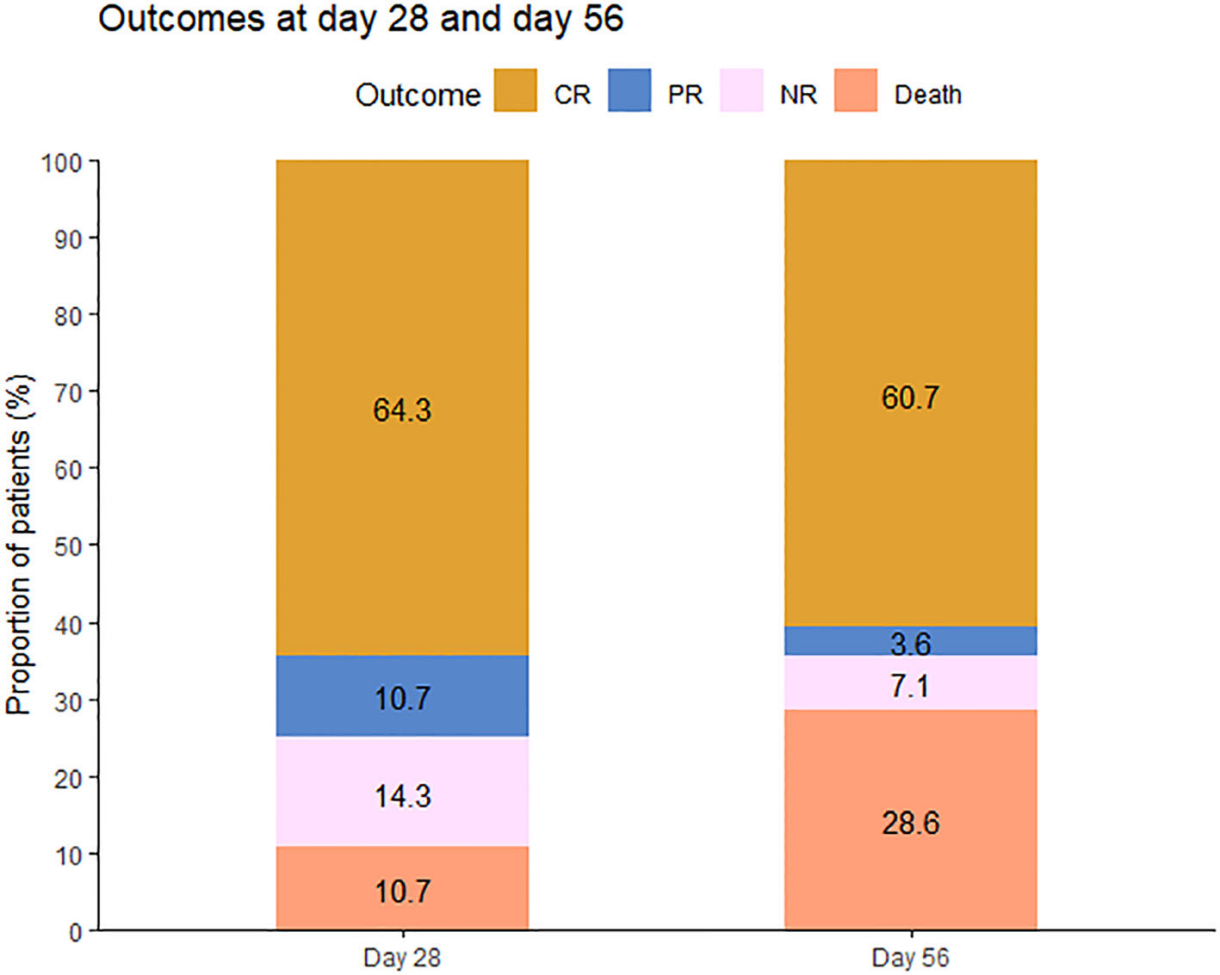
Outcomes at 28 days and 56 days after the initiation of therapy. CR, complete response; PR, partial response; NR, no response.

The primary endpoint, overall response (OR) at day 28, was observed in 21 patients, producing an ORR of 75.0% (95% CI: 54.8%–88.6%). Among the 21 responders, 18 achieved CR (64.3%, 95% CI: 44.1%–80.7%), and three achieved PR (10.7%, 95% CI: 2.8%–29.4%); six (21.4%, 95% CI: 9.0%–41.5%) had no response, two (7.1%, 95% CI: 1.2%–25.0%) of whom died on day 28. The ORR at any time was 78.6% (95% CI, 58.5%–91.0%; CR, 71.4%), which included one patient who had a CR but died of infection before day 28 (**Figure 1**).

### 3.3 Duration of treatment and time to response

**Figure 2:**
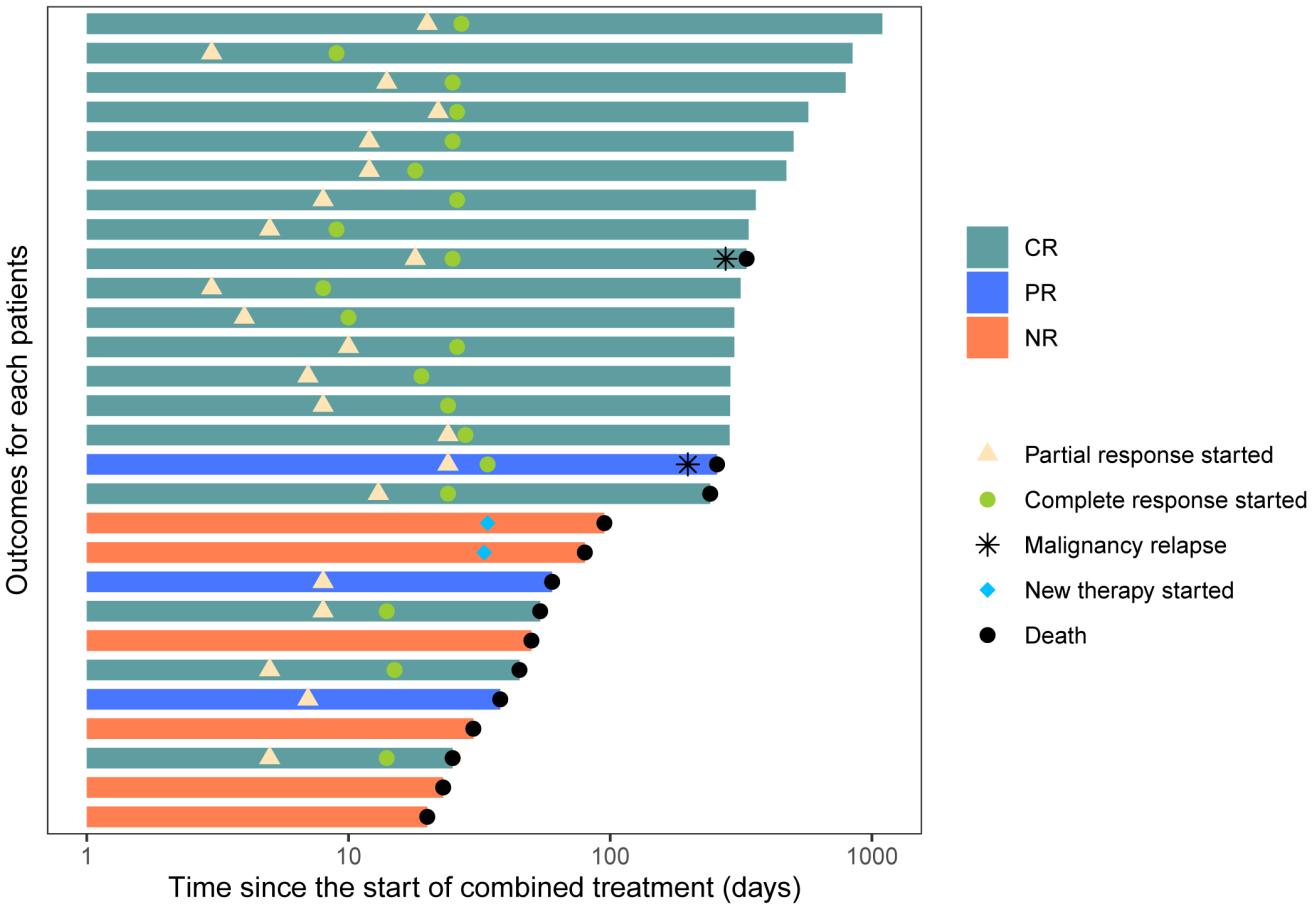
Swimmer plot of therapies received during combination treatment and patient outcomes following initiation of combination treatment.

At day 56, a durable OR was observed in 18 of 28 patients (64.3%, 95% CI: 44.1%–80.7%) and a CR in 17 of 28 patients (60.7%, 95% CI: 40.7%–77.9%). At day 100, 17 of these patients experienced a durable response and were free of LGI-GVHD. Among evaluable patients (n = 28), the median time to PR after starting basiliximab and vedolizumab was 8 days (3–24 days), and the median time to CR was 24 days (8–34 days) (**Figure 2**).

**Figure 3:**
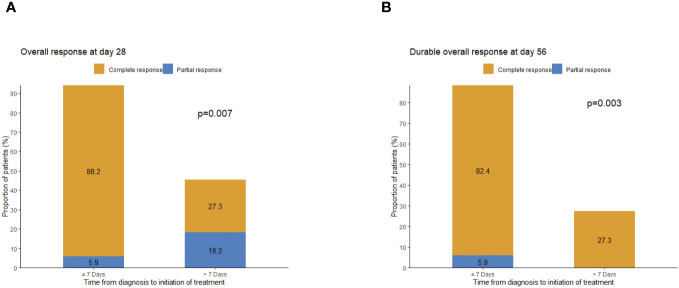
Assessment of response by administered time (start combination treatment ≤7 days and start combination treatment >7 days). **(A)** Overall response at day 28. **(B)** Durable overall response at day 56.

Subgroup analysis of patients’ characteristics showed that day 28 response and durable OR (DOR) were associated with the onset time of treatment after GI-aGVHD was diagnosed. Patients (n = 17) who started treatment of basiliximab and vedolizumab within 7 days after the diagnosis of GI-aGVHD had a statistically significant difference in day 28 OR of 94.1% vs. 45.5% for patients (n = 11) who started after 7 days (p = 0.007). Durable OR at day 56 was 88.2% vs. 27.3% among patients who started treatment within 7 days and later initiation (p = 0.003), respectively (**Figure 3**). It is worth mentioning that there was no significant difference in baseline characteristics between the two groups.

### 3.4 OS, FFS, relapse, and NRM

**Figure 4:**
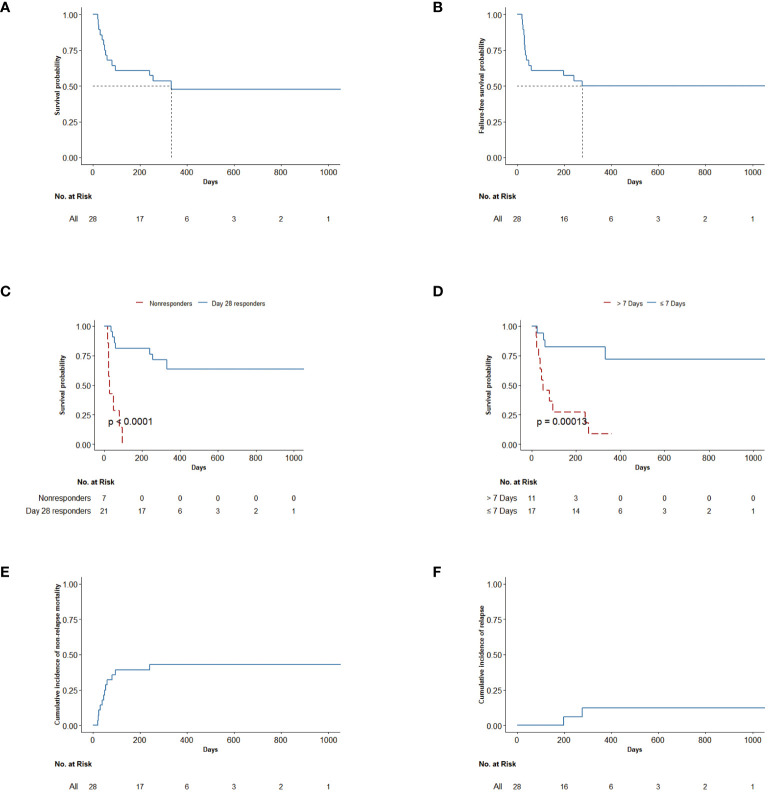
**(A)** OS up to last follow-up after the first combination treatment. **(B)** Failure-free survival of all patients. Failure-free survival was defined as time from first combination treatment to relapse, non-relapse-related death, or the addition of new systemic therapy for aGVHD. **(C)** OS in patients by response status (day 28 responders and non-responders). OS was defined as the time from first combination treatment to death or the date of last follow-up. **(D)** OS in patients by administered time (start combination treatment ≤7 days and start combination treatment >7 days). OS was defined as the time from first treatment to death. **(E)** Cumulative incidence of non-relapse mortality after the first combination treatment. **(F)** Cumulative incidence of relapse after the first combination treatment. OS, overall survival; aGVHD, acute graft-versus-host disease.

The 100-day, 6-month, and 12-month OS rates for the entire cohort of patients were 60.7% (95% CI: 45.1%–81.8%), 60.7% (95% CI: 45.1%–81.8%), and 47.6% (95% CI: 31.4%–72.1%), respectively. The median OS was 332 days (95% CI: 80–not evaluable) (**Figure 4A**). The median failure-free survival was 276 days (95% CI: 50–not evaluable) (**Figure 4B**). For patients in CR or PR (n = 21) at day 28, OS was greater for day 28 responders compared with non-responders (p < 0.0001) (**Figure 4C**). Administered time was also significantly associated with OS. OS was also greater for patients who started treatment of basiliximab and vedolizumab within 7 days compared with later initiation (p = 0.00013) (**Figure 4D**). There was no significant difference in ORR or survival between centers. By 12 months after basiliximab and vedolizumab initiation, two patients died of underlying disease relapse. Twelve patients (34.6%) died of causes other than malignancy relapse, including five (15.4%, 95% CI: 6.8%–37.6%) patients who died of serious infection, four (14.3%, 95% CI: 4.7%–33.6%) who died of progression of aGVHD, and three (10.7%, 95% CI: 2.8%–29.4%) who died of thrombotic microangiopathy (TMA). The 12-month NRM was 42.9% (95% CI: 24.1.0%–60.3%) (**Figure 4E**). Meanwhile, malignancy relapse was observed in two patients (12%, 95% CI: 1.9%–32.8%) at 1-year follow-up (**Figure 4F**).

### 3.5 Adverse events

Basiliximab and vedolizumab did not cause any infusion-related toxicity, hepatotoxicity, or PML in our study. There were no patients in our study who discontinued due to adverse events other than GVHD non-response. Fifty infections were recorded in 20 patients, of which 27 were bacterial, 18 were viral, and five were fungal (**Table 2**). Nine (32.1%) patients developed CMV reactivation complicated with bacterial infections (25.0%, CMV infection; 7.1%, CMV viremia). EBV occurred in five patients (17.9%), and there were no posttransplant lymphoproliferative disorders in our case. Only three patients (10.7%) developed pseudomembranous colitis. Pulmonary infection (21 of 50) was the most common in nine patients. Six (21.4%) patients had coexisting intestinal infections (10 of 50 infections).

**Table 2:** Infections after treatment.

Infections	Number (50)
Viral infections	18
CMV	9
EBV	5
Others	4
Fungal infections	5
Bacterial infections	27
Clostridium difficile	3

CMV, cytomegalovirus; EBV, Epstein–Barr virus.

### 3.6 cGVHD

Among the 17 patients who survived for more than 100 days, cGVHD occurred in seven patients. The 1-year cumulative incidence of cGVHD was 35.3%, and 11.8% of patients developed moderate-to-severe cGVHD.

## 4 Discussion

This retrospective study demonstrated that vedolizumab plus basiliximab for SR-GI-aGVHD patients achieved great clinical efficacy, with a high OR at day 28 and a highly durable OR at day 56.

While vedolizumab is used for induction and maintenance of remission in IBD, its effect on GI-aGVHD remains controversial. Outcomes of the earliest case series reported of vedolizumab in GI-GVHD are variable. Yngvar (18) first reported great outcomes for six patients with SR-GI GVHD after treatment with vedolizumab. All patients exhibited clinical response, and four patients were alive at the last follow-up. However, another data report that included five patients had opposite clinical outcomes, with only two patients achieving partial remission, and all five patients died (16). Within 1 month of starting vedolizumab in the nine patients reported by Alexander (20), a response was observed in eight patients. However, seven of the eight patients who responded died at 2 months of follow-up. However, this case series was limited by small samples. Among the three large retrospective case series that included over 20 patients, over response rate ranged from 35% to 79%, and OS at 6 months ranged from 35.0% to 54% (15, 17, 19). Our study demonstrates that vedolizumab plus basiliximab treatment had an ORR of 75% at day 28 and that patients who responded had prolonged OS compared with non-responders. In contrast to other recently published data, the survival outcomes in our study are acceptable.

What leads to different clinical outcomes of vedolizumab for aGVHD? Potential differences between this study and previous studies related to the combination of nature and inclusion criteria of the current study, together with subtle differences in administration time. As far as we know, this was the first research attempt to investigate vedolizumab plus basiliximab as second-line therapy for SR-GI-aGVHD. Currently, we have several therapy strategies that could be used for SR aGVHD, such as ruxolitinib, monoclonal antibodies, MTX, MSCs, and mTOR inhibitors (21, 24, 25). However, there is little reliable information to determine which agents may be best for SR-GI-aGVHD patients. Ruxolitinib is the first drug approved for SR aGVHD treatment, but it only had 13% CRR and 44.5% ORR on day 28 for SR-GI-aGVHD (26). Basiliximab (anti-CD25 antibody) is also one of the commonly used SR aGVHD treatments that had a response of 65.7% at day 28 (23). It can be found that our approach was comparable with or even better than the current second-line treatment. In contrast, due to the complex pathophysiology of GVHD including multiple molecular mechanisms and multiorgan involvement, blocking integrin alone may not be enough, which may explain why vedolizumab did not meet the primary endpoint in the phase 2a study for SR-GI-aGVHD (27). In our cohort, 24 (85.7%) involved liver aGVHD, and 14 (50.0%) involved skin aGVHD. After combined treatment, most patients had improved. Considering the selectivity of vedolizumab, these may be attributed to basiliximab. Currently, drug combinations lead to a great improvement in efficacy in many reports. As our previous studies reported, MSCs plus basiliximab and calcineurin inhibitor for SR-GI-aGVHD resulted in a response rate of 82.8% by day 28 (28), and fecal microbiota transplantation combined with ruxolitinib also had a high efficacy of 71.4% for GI GVHD (29). In IBD, the combination of vedolizumab and infliximab achieved excellent remission. However, the application of infliximab in GVHD indeed carries a relatively high infection rate (30). The use of a basiliximab–infliximab combination for the treatment of severe GI-aGVHD also yielded unsatisfactory outcomes, with an overall response rate of 76%, while survival at 1 year was only 24%. This outcome seems to be worse than the outcome reported for basiliximab alone (31).

In addition, we also found that earlier administration led to a higher response on day 28 and day 56. Earlier use of the combination was also associated with prolonged OS. However, the best administration time remains unclear. In a retrospective analysis (15) including 29 patients with SR-GI-aGVHD treated with vedolizumab, vedolizumab performed better ORR as a more upfront treatment (13/13 (100%) versus 10/16 (63%)). Azada’s (32) study included pediatric and young adult patients who received vedolizumab and found vedolizumab to be more efficacious and safer when applied early. Ya-Yuan Fu et al. (12) emphasized the crucial role played by the α4β7–MAdCAM-1 interaction in the early recruitment of donor T cells to the intestinal stem cell compartment. If sufficient tissue injury occurs, T-cell migration to the GI endothelium is of limited importance, and α4β7 integrin is no longer required for the propagation of aGVHD. In a phase 1b, open-label, dose-finding study, vedolizumab showed a low incidence of GI-aGVHD and grade III to IV aGVHD when added to GVHD prophylaxis (14). These suggested to us that earlier treatment with vedolizumab may be beneficial for prognosis.

Regarding safety, we found that vedolizumab plus basiliximab did not increase the side effects of infections. Several studies have demonstrated that vedolizumab or basiliximab was safe for patients with SR-GI-aGVHD, even for pediatric patients (32–34). In addition, the combination therapy was well-tolerated, and there was no infusion-related response in our study. Three patients developed TMA, but it occurred after the end of combination treatment. The rate of infection was 71.4% in our study, and six (23.1%) patients had coexisting intestinal infections. CMV reactivation occurred in 26.9% of cases, and EBV infection occurred in 18.2% of patients. Infections in our cohort were similar to those in other studies. In addition, our patient had a relatively low incidence of *Clostridium difficile* infection, which may be attributed to preventive vancomycin usage. However, these infections could not be clearly attributed to vedolizumab because of its gut-selective mechanism and the previous glucocorticoid for GI aGVHD.

There are several limitations to our study. First, although it was the first study of vedolizumab combined with basiliximab as a second-line treatment for SR-GI-aGVHD, it is a small retrospective study and lacks a control group. Second, we did not check the expression of α4β7 integrin on T cells. Finally, vedolizumab was administered according to the dose and schedule for treatment of IBD, which is possibly inappropriate. Future studies for programmed administration and individualized dosing based on aGVHD population are warranted.

## 5 Conclusion

In summary, this study showed that vedolizumab plus basiliximab therapy leads to higher therapeutic response and prolonged OS of SR-GI-aGVHD patients and is well-tolerated. Vedolizumab plus basiliximab may be considered a potential treatment option for LGI-aGVHD patients.

## Data availability statement

The raw data supporting the conclusions of this article will be made available by the authors, without undue reservation.

## Ethics statement

The studies involving humans were approved by Ethics Committee of Southern Hospital of Southern Medical University. The studies were conducted in accordance with the local legislation and institutional requirements. The participants provided their written informed consent to participate in this study. Written informed consent was obtained from the individual(s) for the publication of any potentially identifiable images or data included in this article.

## Author contributions

ZG: Writing – original draft, Writing – review & editing. ZF: Writing – original draft, Writing – review & editing. ZL: Writing – review & editing. XY: Writing – review & editing. YZ: Writing – review & editing. LX: Writing – review & editing. FH: Writing – review & editing. RL: Writing – review & editing. JS: Writing – review & editing. QL: Writing – review & editing. NX: Writing – original draft, Writing – review & editing.

## Glossary

**Table:**

GVHD	graft-versus-host disease
aGVHD	acute GVHD
allo-HSCT	allogeneic hematopoietic stem cell transplantation
GI	gastrointestinal
SR	steroid-resistant
MAdCAM-1	mucosal addressing cellular adhesion molecule1
IBD	inflammatory bowel disease
IL-2R	interleukin-2 receptor
MTX	methotrexate
mTOR	mammalian target of rapamycin
MSCs	mesenchymal stromal cells
FMT	fecal microbiota transplantation
CR	complete response
NR	no response
PR	partial response
OR	overall response
ORR	overall response rate
DOR	durable OR
OS	overall survival
AEs	adverse events
CMV	cytomegalovirus
EBV	Epstein–Barr virus
NRM	non-relapse mortality
cGVHD	chronic GVHD
FFS	failure-free survival
OS	overall survival
CsA	cyclosporine A
MMF	mycophenolate mofetil
PML	progressive multifocal leukoencephalopathy
